# Early career researchers and mental health: Observational study of challenge and wellbeing

**DOI:** 10.1002/hsr2.1649

**Published:** 2023-11-20

**Authors:** Eleonora Cilli, Jessica Ranieri, Federica Guerra, Dina Di Giacomo

**Affiliations:** ^1^ Department of Life, Health and Environmental Sciences University of L'Aquila L'Aquila Italy; ^2^ Postgraduate School on Clinical Psychology University of L'Aquila L'Aquila Italy

**Keywords:** early research careers, emotional regulation, mental health, researcher skills, stress

## Abstract

**Background:**

Early career researchers (ECRs) are a strategic sector in the academic community because they represent a scientific incubator for future academic scholars. Recently, growing evidence suggests that relevant doctoral researchers work under elevated levels of stress and frustration and that this has a significant impact on their personal health and research output and their future career development. This study aimed to analyse the well‐being and mental health within ECR, focusing on coping strategies for stress, and to contribute and exploit a conceptual framework tailored to the academic context considering the specifics and challenges of academia.

**Methods:**

Participants were 134 young early career academics (mean age = 30.6; *SD* = 4.38; range = 25–40 years) enrolled via institutional email. A 94‐item questionnaire was created using Survey Monkey and distributed between October and December 2022. The survey assessment was based on three parts: (1) sociodemographic data, (2) psychological assessment, and (3) research skills design.

**Results:**

Our findings highlighted a general trend toward negative psychological dimensions in ECRs: PhD students and research contracts appeared to be stressed, anxious, and depressed. Moreover, they had segmented ECRs: PhD students showed higher levels of depression, anxiety, and stress than research contracts, highlighting reduced positive outcomes in psychological dimensions, as well as lower ability to manage emotional experiences and then to be perseverant for long‐term goals and motivation. Our findings highlight that mental health in ECRs is a challenge that needs to be addressed in academia.

**Conclusions:**

New and innovative ways of encouraging help‐seeking must be developed and implemented to address policy changes, communication activities, training, and health‐promotion activities through the circulation of experience, sharing actions, and strategies to foster healthy academics by raising awareness, implementing interventions, or engaging professionals concerning mental health in academia.

## 1 INTRODUCTION

Early career researchers (ECRs) are a strategic sector of the academic community because they represent a scientific incubator for future academic scholars. Recently, the mental well‐being and health of researchers and employees in academia have been increasing.^1^, ^2^, ^3^ Peterson et al.^3^ highlighted that understanding how institutional changes within academia may affect the overall potential of science requires a better quantitative representation of how careers evolve.^4^ Some recent rearranging in academia included the changing working structure of research universities, a reduction in the number of tenure‐track positions, and a related policy shift away from long‐term contracts.^5^ The focal point of academia changing over time is the negative impact of professions designed around short‐term contracts related to implicit expectations of sustained annual production that effectively discount the cumulative achievements of the individual. Furthermore, short‐term contracts might reduce young scientists’ incentives to invest in human and social capital accumulation.^2^ Moreover, the importance of the employment relationship could combine positive competitive pressure with adequate safeguards to protect against career hazards and endogenous production uncertainty that an individual is likely to encounter in his/her career. The evaluation of research productivity of researchers, faculty, academic qualifications, and experience for the purposes of recruitment, promotion, and research grant funding represents salient experiences that could be focused on emotional dynamics based on stress, psychological distress, anxiety, and depression.^6^ The young researcher study underlined the above scenario: the individual contribution to build academic professional could have a relevant impact.^7^

Recently, a growing body of evidence suggests that relevant doctoral researchers work under elevated levels of stress and frustration, which significantly impacts their personal health and research output and their future career development.^8^, ^9^, ^10^ This study aimed to analyse the well‐being and mental health within ECR, focusing on coping strategies for stress, and to contribute and exploit a conceptual framework tailored to the academic context considering the specifics and challenges of academia. We aimed to investigate the psychological dimensions of early career academics in academia and draw on well‐being in the scientific environment in an attempt to detect the need for research skills in terms of scientific skills and networking.

## 2 MATERIALS AND METHODS

### 2.1 Ethical approval

This study was approved by the Institutional Review Board (IRB) of the University of L'Aquila (Code 44/2022). Signed informed consent, based on the Declaration of Helsinki^11^ was mandatory.

### 2.2 Participants

Participants were 134 young ECRs (mean age = 30.6; *SD* = 4.38; range = 25–40 years) who were enrolled via institutional email. A 94‐item questionnaire was created using Survey Monkey and distributed between October and December 2022. The inclusion criteria were as follows: (a) age range 25–40; (b) being included in advanced research training (PhD student, research fellow, research contract) in academia; and (c) written informed consent. Participation in this study was voluntary. Those who did not meet any of the inclusion criteria were excluded from participation in the survey using the gated question method.

### 2.3 Measures

The survey assessment was based on three parts: (1) sociodemographic data, (2) psychological assessment, and (3) research skills design.

#### 2.3.1 Sociodemographic and individual characteristics

The sociodemographic characteristics of the participants, age, sex, civil status, academic roles (PhD student, research fellow, research contract), scientific fields by European Research Council categorization (ERC) [Life Sciences (LF), Physical & Engineering (PE), Social Humanities (SH)], and years of employment (>12 months, 12–24 months, 24–36 months, <36 months) were assessed using a socio‐demographic form.

#### 2.3.2 Psychological assessment

The psychological battery consists of four standardized self‐reports measuring emotional traits (depression, anxiety, and stress), personality dimensions, and trait‐level perseverance. Each standardized test was applied using Italian population adaptation.

*Depression Anxiety Stress Scales 21* (*DASS‐21*)^12^: The DASS‐21 is a self‐report measure of the degree of severity of three emotional indices: depression, anxiety, and stress. It is composed of 21 questions with responses on a 4‐point Likert‐type scale.

*Difficulties in Emotion Regulation Scale‐20* (DERS‐20)^13^: DERS‐20 is a test to assess individual differences in the ability to identify, accept and manage emotional experience; the test is composed of n. 5 indexes: (a) Nonacceptance, (b) Goals, (c) Impulse, (d) Awareness, (e) Strategies. Nonacceptance is an index that evaluates nonacceptance of emotional responses, better the tendency to experience negative secondary emotions in response to a negative emotion or to demonstrate a reaction of nonacceptance concerning one's discomfort; the Goals index detects the difficulties engaging in goal‐directed behavior, and it reflects difficulties in concentrating and performing a task when experiencing negative emotions; impulse index is oriented to impulse control difficulties, and detects the difficulty in maintaining control when one feels negative emotions; the awareness index is based on the lack of emotional awareness, and it identifies the tendency to pay attention to emotions and the relative ability to recognize them. Finally, the strategies index deals with limited access to emotion regulation strategies and reflects the belief that it is particularly difficult to regulate emotions once they have occurred. The score is obtained by standard scoring, with a higher score indicating greater difficulty in emotion regulation.

*Short Grit Scale* (*Grit‐S*)^14^: It is a self‐report questionnaire which measures trait‐level perseverance and passion for achieving long‐term goals. Each item is endorsed using a 5‐point Likert‐type scale, which provides a total score and two subscale scores: Perseverance of Effort and Consistency of Interest. These subscales evaluate interest in tasks, ability to respond to setbacks, consistency in distraction, engagement, ability to complete goals, and conscientiousness.

#### 2.3.3 Research skills designing

Research skills have been evaluated by an ad hoc questionnaire built to evaluate the scientific skills and networking needs to detect the needs of the ECRs generation.

*Scientific skills*: Scientific skills were analysed to detect the need for advanced education. The skills investigated were: (1) Interpretation of data, graphs, and tables; (2) Problem solving‐critical thinking; (3) Ability in the discussion of data; (4) Advanced statistical analyses; (5) Hypothesizing: verifiable, measurable, and repeatable; (6) Understanding of hypothesis null and alternative; (7) Design of experiment: identifying and controlling variables; (8) Observing and comparing competence in describing patterns; (9) Reviewing and communicating information; (10) Ability to test the hypothesis; (11) Predictive analyses; (12) Creating representative graphs; (13) Grouping and organizing topics; and (14) Accuracy and precision measurement.

*Networking*: Additional items investigated self‐perception of networking academy activities. They were asked to indicate the degree of usefulness of the activities, such as (a) interdisciplinary project days; (b) university research workshops; (c) career days; (d) outdoor activities; (e) Researcher Toolkit training; (f) soft skills lab; (g) mutual aid groups; (h) yoga sessions; (i) mindfulness; (j) psychological counseling; and (k) psychotherapy.

### 2.4 Survey instruments

The survey instrument combined demographic and career status questions. Additionally, respondents were asked about their stress, anxiety, and emotional distress using validated measures (see Section 2.3).

### 2.5 Procedure

Participants were enrolled via institutional email by the Psychological Counseling of Laboratory of Clinical Psychology (Prof. Dina Di Giacomo), Department of Clinical Medicine, Public Health, Life and Environmental Sciences (MeSVA), University of L'Aquila (IT). Informed consent was obtained from all participants.

### 2.6 Study design

An observational study was conducted on early carrier researchers. Descriptive statistics (mean, standard deviation, percentage), one way analysis of variance (ANOVA), multivariate analysis of variance (MANOVA) and, normality test (Shapiro–Wilk test) were used to examine the variables. Partial correlation analysis (post hoc analysis) was conducted to examine the relationship between all variables. All statistical analyses were carried out using Jamovi Statistics software program.^15^ The significance level was fixed at *α* < 0.05.

## 3 RESULTS

**Figure 1:**
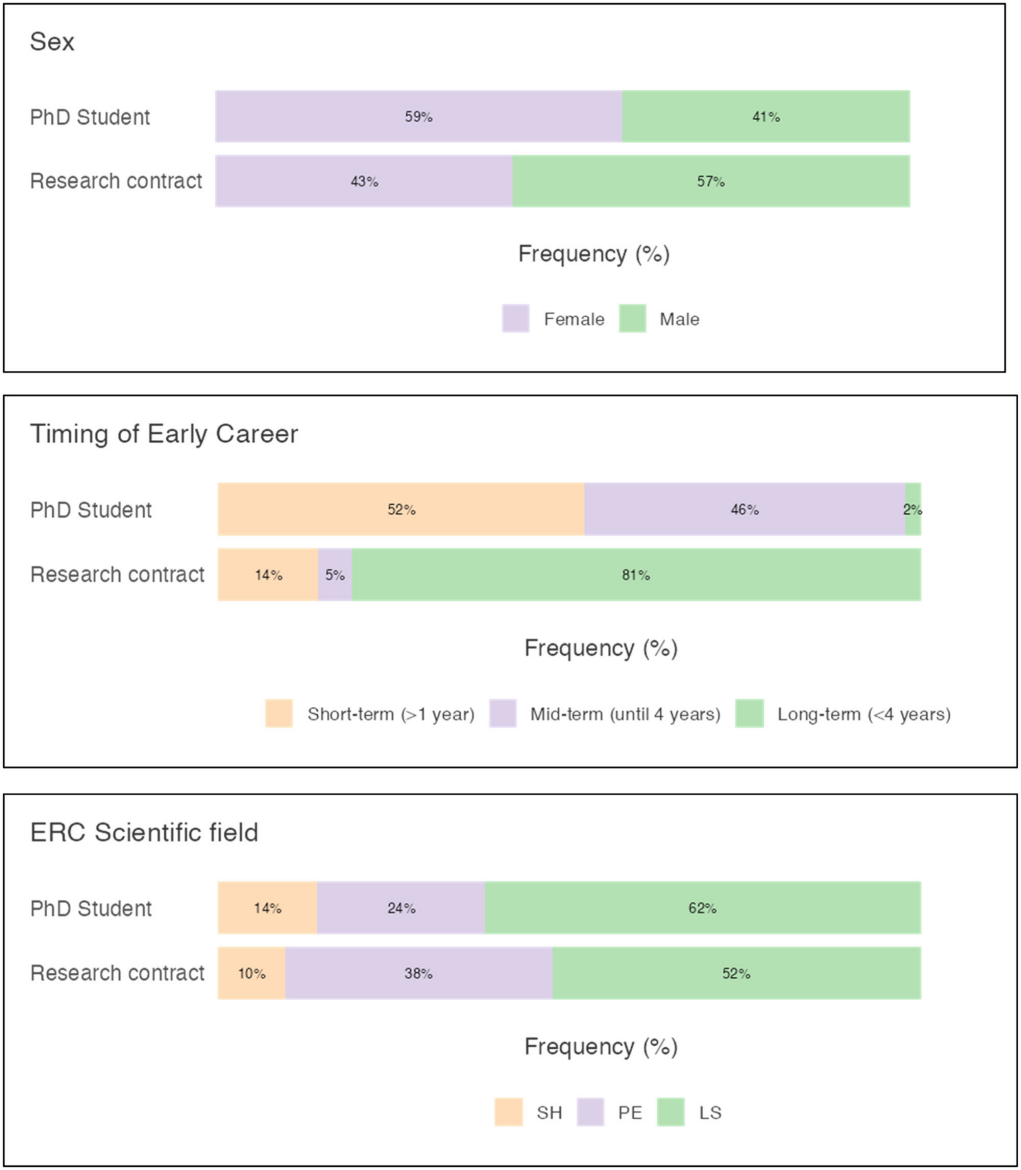
Representation of distribution by sex, academic roles, timing of early career.

Table 1 reports the sociodemographic data of the participants, whereas Figure 1 shows the distribution of the sample by sex, timing of early career, and ERC scientific field.

**Table 1:** Sociodemographic data of participants.

	Early career researchers (*n* = 134)
Age (years)	X30.6 *SD* ± 4.38 (min. = 25; max. = 40)
Gender: *n* (%)	
Female	72 (53.7%)
Male	62 (46.3%)
Academic role: *n* (%)	
PhD students	92 (68.7%)
Research contract	42 (31.3%)
Marital status: *n* (%)	
Single	84 (62.7%)
Engaged	44 (32.8%)
Prefer not to say	6 (4.5%)
ERC scientific field: *n* (%)	
Life Sciences (LS)	79 (59.0%)
Physical & Engineering Sciences (PE)	38 (28.4%)
Social Humanities and Sciences (SH)	17 (12.7%)
Timing of early career: *n* (%)	
Short‐term (>1 year)	54 (40.3%)
Mid‐term (until 4 years)	44 (32.8%)
Long‐term (<4 years)	36 (26.9%)

The study sample comprised 134 participants distributed into two ECR groups: PhD students (*n* = 92) and research contracts (*n* = 42). The distribution into two groups reflects the ongoing reduction of academic position and the changing for working applying.

### 3.1 Psychological patterns

Participants were evaluated using standardized psychological tests focused on measuring (a) emotional dimensions (depression, anxiety, and stress), (b) the individual's ability to identify, accept, and manage emotional experience, and (c) the ability to maintain focus and interest, and persevere in obtaining long‐term goals. Table 2 reports the mean values and standard deviations of the sample for standardized psychological testing.

**Table 2:** Raw scores of psychological evaluations.

			Shapiro–Wilk	
	Mean	*SD*	*W*	*p*
DASS‐21 test				
Depression	11.57	10.93	0.88	<0.001
Anxiety	5.57	5.72	0.86	<0.001
Stress	15.57	9.99	0.96	0.003
DERS‐20 test				
Nonacceptance	10.23	5.28	0.88	<0.001
Goals	10.72	4.71	0.93	<0.001
Impulse	6.81	3.99	0.75	<0.001
Strategies	6.34	2.87	0.89	<0.001
Awareness	10.51	3.81	0.96	<0.001
GRIT‐S test				
Consistent of interest	3.67	0.87	0.92	<0.001
Perseverance of effort	3.70	0.73	0.96	0.003

Abbreviations: DASS‐21, Depression Anxiety Stress Scales 21; DERS‐20, Difficulties in Emotion Regulation Scale‐20; Grit‐S, Short Grit Scale.

First, we wanted to detect the emotional dimensions of early academic researchers; MANOVA (3 × 2 × 3) was conducted to compare the DASS‐21 indexes (3: depression, anxiety, stress) to academic roles (2: PhD student; research contract) and ERC scientific fields (3: SH, LS, PE). The comparison of academic roles into three emotional dimensions emerged as significant (*F*_(126, 3)_ = 2.76; *p* = 0.04); there were no significant differences in the ERC scientific field and no interaction. The following one‐way ANOVA (3 × 2) on emotional dimensions between academic roles showed that PhD students suffered from higher depression, anxiety, and stress than research contracts (see Table 3).

**Table 3:** One‐way ANOVA and post hoc test for academic role in DASS‐21 variables.

	Academic role	*N*	Mean	*SD*	*SE*	*F*	*p*	Post hoc test *p*
Depression score	PhD student	92	13.22	11.92	1.243	9.86	0.002	0.000***
	Research contract	42	7.95	7.29	1.125			
Anxiety score	PhD student	92	6.41	5.99	0.625	8.10	0.005	0.005**
	Research contract	42	3.71	4.62	0.713			
Stress score	PhD student	92	17.02	10.85	1.131	8.92	0.003	0.003**
	Research contract	42	12.38	6.90	1.065			

*Note*: *p < 0.05; **p < 0.01; ***p < 0.001.

Abbreviations: ANOVA, analysis of variance; DASS‐21, Depression Anxiety Stress Scales 21.

We then processed the data to analyse the ability to manage emotional experiences. Table 4 reports the statistical analysis results.

**Table 4:** One‐way ANOVA and post hoc test for academic role in DERS variables.

	Academic role	*N*	Mean	*SD*	*SE*	*F*	*p*	Post hoc *p*
Nonacceptance	PhD student	92	10.57	5.28	0.550	1.173	0.28	0.28
	Research contract	42	9.50	5.29	0.816			
Goals	PhD student	92	11.27	4.80	0.500	4.04	0.04*	0.04*
	Research contract	42	9.52	4.36	0.672			
Impulse	PhD student	92	7.52	4.50	0.469	10.0	0.002*	0.002**
	Research contract	42	5.24	1.81	0.279			
Strategies	PhD student	92	6.55	2.83	0.295	1.70	0.19	0.19
	Research contract	42	5.86	2.95	0.455			
Awareness	PhD student	92	10.41	3.60	0.376	0.20	0.64	0.64
	Research contract	42	10.74	4.29	0.662			

*Note*: *p < 0.05; **p < 0.01; ***p < 0.001.

Abbreviations: ANOVA, analysis of variance; DERS, Difficulties in Emotion Regulation Scale.

MANOVA (5 × 2 × 3) was conducted to compare the DERS indexes (5: Nonacceptance, Goals, Impulse, Awareness, Strategies) into academic roles (2: PhD student; research contract) and ERC scientific fields (3: SH, LS, PE). The comparison of academic roles into three emotional dimensions emerged as significant (*F*_(126, 3)_ = 2.76; *p* = 0.04); there were no significant differences in the ERC scientific field and no interaction (see Table 4). One‐way ANOVA revealed higher difficulties in Goals and Impulse indexes for PhD students than for research contracts (see Table 5).

**Table 5:** One‐way ANOVA and post hoc test for academic role in DERS variables.

	Academic role	*N*	Mean	*SD*	*F*	*p*	Post hoc *p*
Consistent of interest	PhD student	92	3.58	0.929	4.66	0.03	0.05
	Research contract	42	3.89	0.690			
Perseverance of effort	PhD student	92	3.60	0.750	6.93	0.01	0.01*
	Research contract	42	3.93	0.656			

*Note*: *p < 0.05; **p < 0.01; ***p < 0.001.

Abbreviations: ANOVA, analysis of variance; DERS, Difficulties in Emotion Regulation Scale.

Finally, we examined the ability to persevere for long‐term goals. MANOVA 2 × 2 × 3 was conducted to compare the GRIT indexes (2: Perseverance of Effort, Consistency of Interest) into academic roles (2: PhD student; research contract) and ERC scientific field (3: SH, LS, PE); statistical analysis showed significant differences between academic roles, no significant difference between ERC field, and no interaction.

One‐way ANOVA comparing GRIT indexes and academic roles highlighted lower persistence traits (*p* = 0.05) and lower consistency of interest (*p* = 0.01) in PhD students than in research contracts (see Table 4).

### 3.2 Research skills challenge

**Figure 2:**
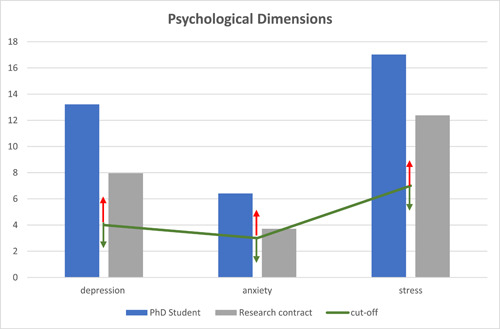
Representation of psychological dimensions of participants.

An ad hoc questionnaire was used to detect qualitative data regarding the need for scientific skills and networking. Participants were asked to highlight the relevance of research skills that need to be enhanced by advanced courses. Figure 2 shows the prevalence of some skills to enhance: interpretation of data/graphs//tables 94%, problem solving‐critical thinking 75%, ability in the discussion of data 63%, and advanced statistical analyses 53%.

**Figure 3:**
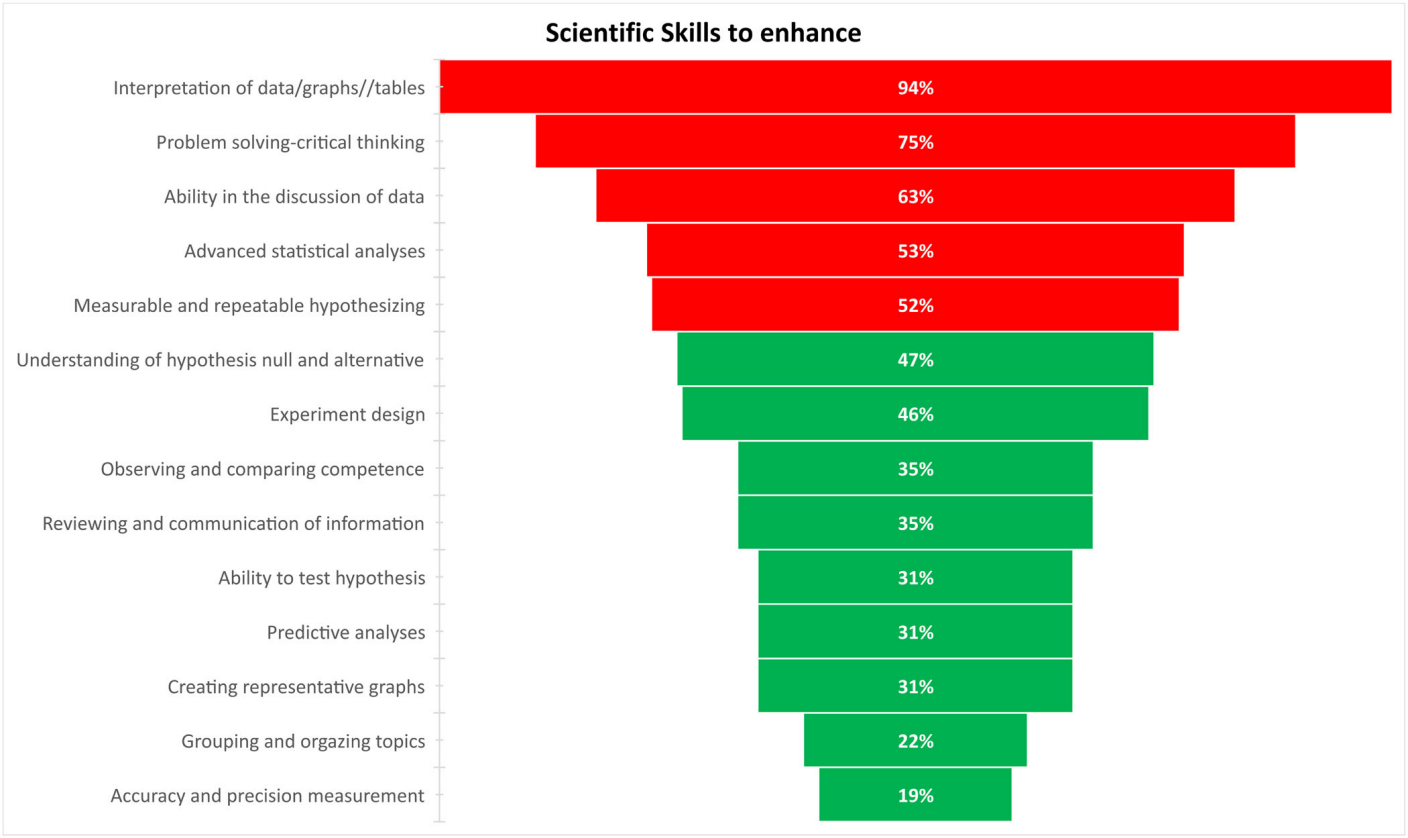
Representation of scientific skills to enhance.

Networking activities (see Figure 3) were grouped into three categories: scientific networking, social networking, and individuals. In scientific networking (see Figure 4), ECRs evidenced the relevance of the need to reinforce the training for researcher toolkits (69%) and the participation of dedicated workshops in university research. Social networking could be reinforced by outdoor (63%) and team building (61%) activities. Finally, the need to boost individual mental health emerged as relevant: psychological counseling (65%), psychotherapy (58%), and soft skills (56%) seemed to be considered useful.

Figure 4Needs of networking activities representation.

**Figure:**
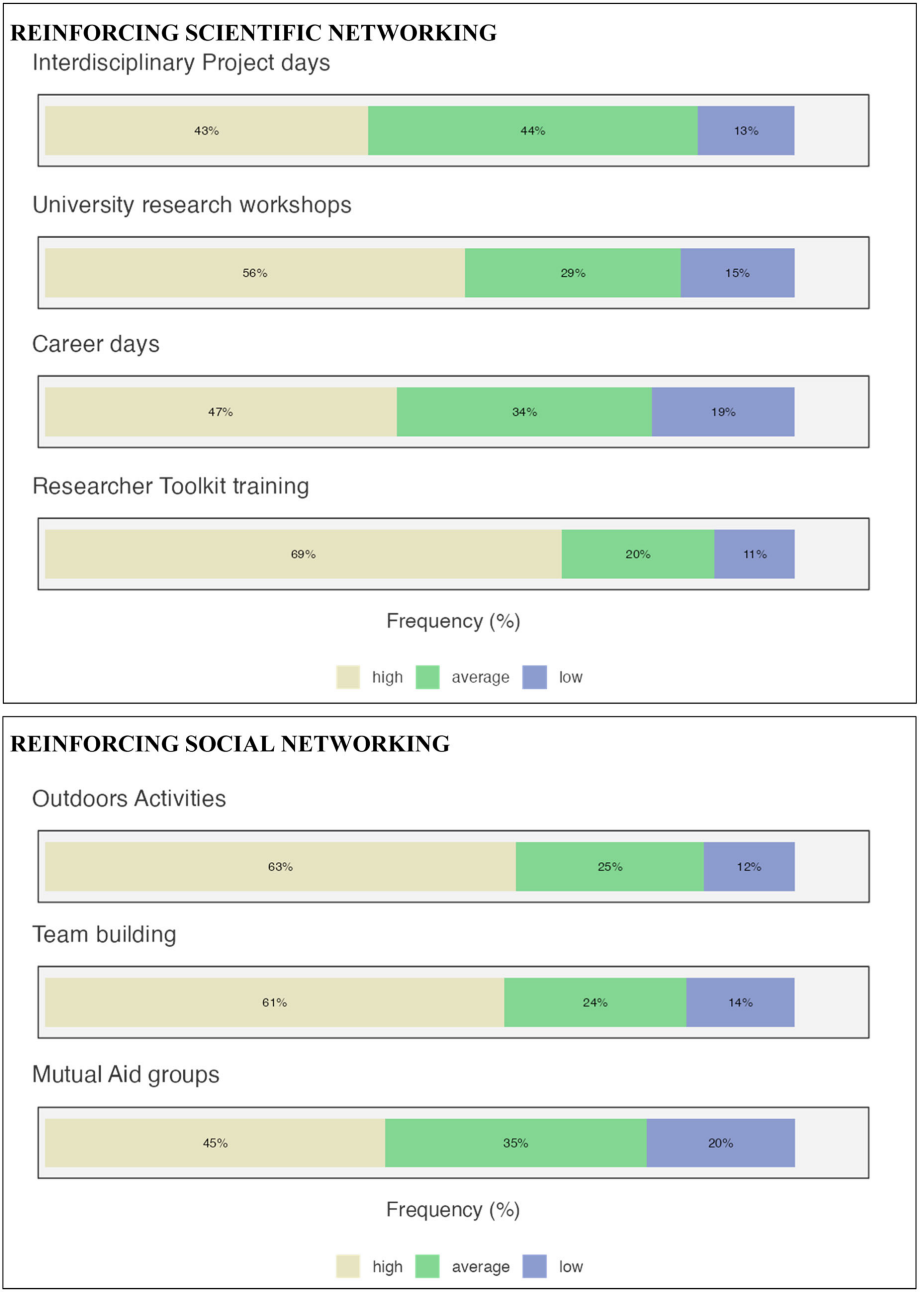

**Figure:**
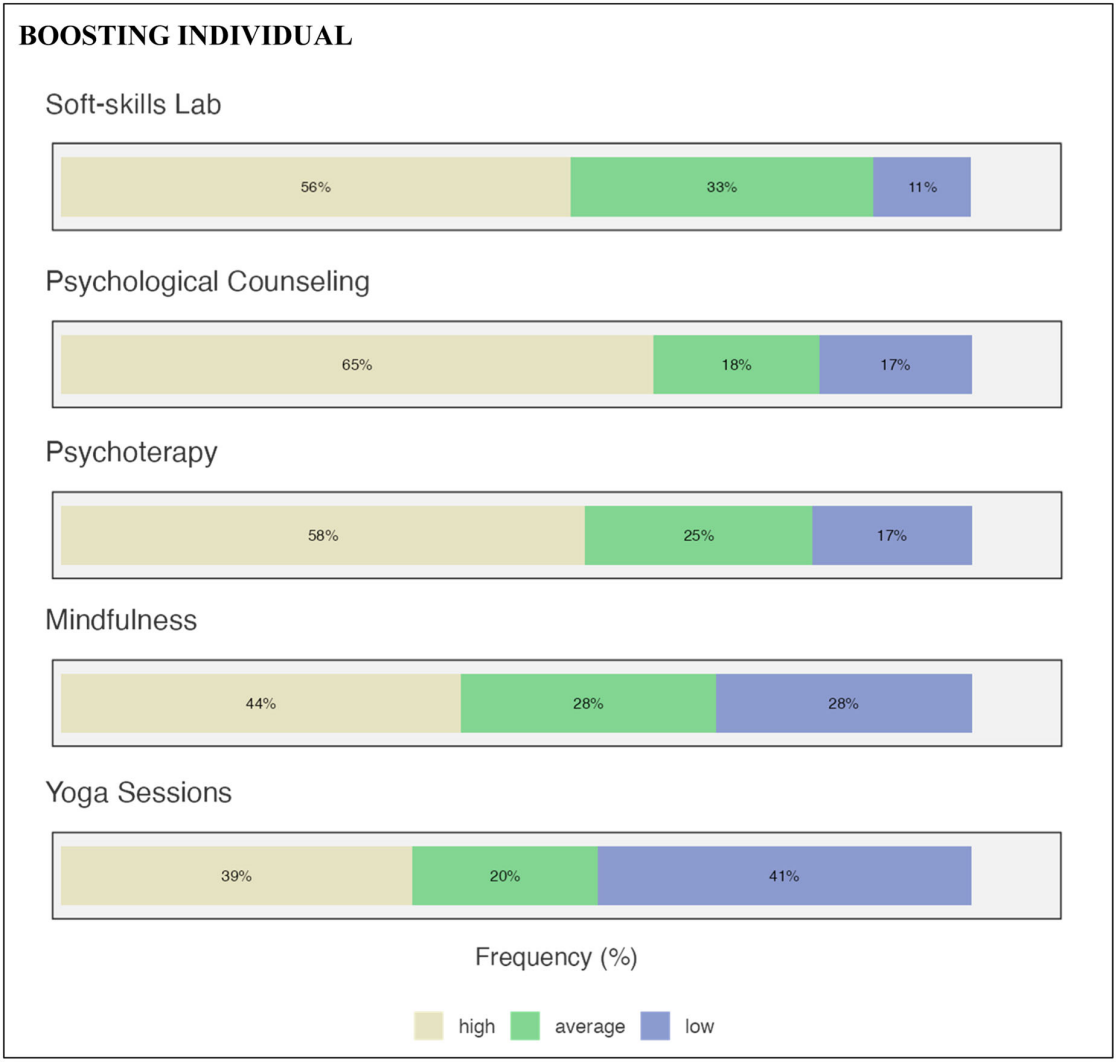

## 4 DISCUSSION

Our study aimed to investigate the mental health of ECRs by analysing psychological dimensions, the ability to manage emotional experience, and the ability to be perseverant for long‐term goals. We drew on the researcher's needs in terms of scientific and networking exploitation. The focus of this study was to contribute to developing a conceptual framework tailored to the academic context, considering the specifics and challenges of academia.

Our findings highlight a general trend toward negative psychological dimensions in ECRs: PhD students and research contracts appeared stressed, anxious, and depressed, as represented in Figure 2, and both groups reported performance overcoming the positive cut‐off. Moreover, they had segmented ECRs: PhD students showed higher levels of depression, anxiety, and stress than research contracts, highlighting reduced positive outcomes in psychological dimensions, as well as lower ability to manage emotional experiences and then to be perseverant for long‐term goals and motivation.

Our findings highlight that mental health in ECRs is a challenge that needs to be addressed in academia. According to the literature, ECRs can be affected by negative psychological dimensions. The added value of our findings relates to our tailored research on ECRs. Guthrie's report^16^ evidenced that the weakness of evidence‐based insight on this topic is related to the prevalence of investigation stress and mental health problems among academics and other university staff^17^ as well as postgraduates.^18^, ^19^ Considering the literature, our study focused on psychological dimensions that depend on the management and self‐regulation of emotions. Findings showed the need for PhD students to improve their commitment to higher‐order goals: behavioral change could positively impact the well‐being of the young and, at the same time, enhance the cumulative achievements of the individual for scientific productivity.

Then, using a bottom‐up approach, we enriched the results to detect whether scientific skills and networking tools would be beneficial. The ECRs were asked to indicate the advanced learning needs they perceived as strengthening scientific skills. Interpretation of data/graphs/tables, problem‐solving, critical thinking, ability in the discussion of data, advanced statistical analyses, and measurable and repeatable hypothesizing have been subjects considered more relevant for their own scientific skills and to deal with in‐depth by advanced learning. Joined scientific skills and the triple helix of networking (scientific, social, and individual) for personal and career growth. Our findings corroborate previous results.^16^

As a result, outreach efforts for ECR mental health are needed to maintain talented scholars in academic settings. In our opinion, the creation of (local and/or local) observatory for the mental health of the researcher could be a useful instrument for policy change corresponding to efforts to change the overall policy related to mental health and well‐being issues and promote mental health and well‐being to academic research staff. New and innovative ways of encouraging help‐seeking must be developed and implemented to address policy changes, communication activities, training, and health‐promotion activities through the circulation of experience, sharing actions, and strategies to foster healthy academics by raising awareness, implementing interventions, or engaging professionals about mental health in academia.^20^

This study has several limitations. First, the low response rate of this nonrandom sample resulted in a highly skewed study population, with more females than males responding. As the goal of the screening was to identify and triage at‐risk ECR, the survey respondents may have represented young people with more severe mental health needs who responded to the survey as a means of seeking help. Second, the study's sample size could be improved, and more sophisticated analyses could be conducted, greatly limiting the generalizability of the study's findings.

## 5 CONCLUSIONS

These findings have major implications for addressing the mental health needs of ECRs that correlate with improvements in research skills. Emotional involvement in the research context represents a challenge for academia: reinforcing young talent means identifying and triaging those at high risk for mental health disorders to activate coping strategies and arrange tailored training.

## AUTHOR CONTRIBUTIONS

**Eleonora Cilli**: data curation; validation; writing—original draft. **Jessica Ranieri**: investigation; writing—review & editing. **Federica Guerra**: formal analysis. **Dina Di Giacomo**: conceptualization; writing—review & editing.

## CONFLICT OF INTEREST STATEMENT

The authors declare no conflicts of interest.

## TRANSPARENCY STATEMENT

The lead author Dina Di Giacomo affirms that this manuscript is an honest, accurate, and transparent account of the study being reported; that no important aspects of the study have been omitted; and that any discrepancies from the study as planned (and, if relevant, registered) have been explained.

## Data Availability

Not available.
